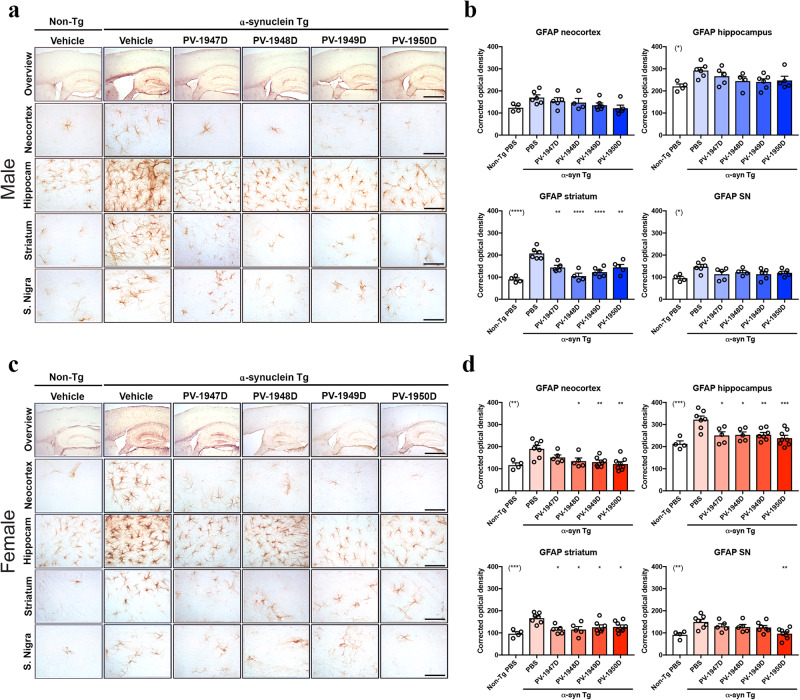# Author Correction: Efficacy and immunogenicity of MultiTEP-based DNA vaccines targeting human α-synuclein: prelude for IND enabling studies

**DOI:** 10.1038/s41541-023-00703-0

**Published:** 2023-07-17

**Authors:** Changyoun Kim, Armine Hovakimyan, Karen Zagorski, Tatevik Antonyan, Irina Petrushina, Hayk Davtyan, Gor Chailyan, Jonathan Hasselmann, Michiyo Iba, Anthony Adame, Edward Rockenstein, Marcell Szabo, Mathew Blurton-Jones, David H. Cribbs, Anahit Ghochikyan, Eliezer Masliah, Michael G. Agadjanyan

**Affiliations:** 1 Laboratory of Neurogenetics, National Institute of Aging, National Institute of Health, Bethesda, MD USA; 2 Department of Molecular Immunology, Institute for Molecular Medicine, Huntington Beach, CA USA; 3 Institute for Memory Impairments and Neurological Disorders, University of California Irvine, Irvine, CA USA; 4 Sue and Bill Gross Stem Cell Research Center, University of California Irvine, Irvine, CA USA; 5 Department of Neurosciences, University of California, San Diego, La Jolla, CA USA

**Keywords:** Parkinson's disease, DNA vaccines

Correction to: *npj Vaccines* 10.1038/s41541-021-00424-2, published online 10 January 2022

In the original version of this Article, images from Fig. 7c column PV-1950D were mistakenly duplicated in Fig. 7a column PV-1950D. The figure has been corrected in both the PDF and HTML versions of this Article.